# Assessing Emergency Medicine, Family Medicine, and ICU Doctors’ Knowledge, Confidence, and Attitude in Managing In-Flight Medical Emergencies in the Kingdom of Saudi Arabia Hospitals: A Cross-Sectional Study

**DOI:** 10.7759/cureus.78359

**Published:** 2025-02-01

**Authors:** Shrooq M Hawati, Fares Binobaid, Asmaa Alsaeigh, Walaa Alameer, Ali M Al Ajmi, Mohammed K Alghamdi, Alwaleed A Alqarni, Saad N Al-Harthi, Abdullah Almelaifi, Abdulrahman A Al Shehri

**Affiliations:** 1 Emergency, Security Forces Hospital-Makkah, Makkah, SAU; 2 Emergency Medicine, King Abdulaziz Hospital-Makkah, Makkah, SAU; 3 Medicine and Surgery, Umm Al-Qura University, Makkah, SAU; 4 Emergency Medicine, King Abdulaziz Medical City-Jeddah, Jeddah, SAU; 5 Medicine and Surgery, King Faisal University, Al Hassa, SAU; 6 Medicine and Surgery, King Faisal University, Dammam, SAU; 7 Medicine and Surgery, King Faisal University, Al Hofuf, SAU

**Keywords:** emergency doctors, emergency medicine physician, family medicine, in-flight emergencies, medical icu, medical managment, saudi hospitals, s: saudi arabia, travel health, cross-sectional studies

## Abstract

Background: In-flight medical emergencies (IFMEs) present unique challenges for healthcare professionals, requiring a specific set of knowledge and skills that are not typically covered in standard medical training. This study aims to assess the knowledge, practices, and confidence levels of healthcare professionals in managing IFMEs, as well as their understanding of aviation physiology.

Methodology: A cross-sectional study was conducted among 5,000 healthcare professionals from various specialties and regions. Participants completed a comprehensive survey assessing their demographic characteristics, clinical knowledge, aviation physiology knowledge, and attitudes toward IFMEs. Data were analyzed to identify knowledge gaps, regional and specialty-based variations, and factors influencing confidence levels in handling in-flight emergencies.

Results: The study revealed that 3745 (74.9%) of the participants demonstrated adequate knowledge, with significant variations observed across regions and specialties. Intensive care unit (ICU) specialists and participants from the Middle region showed the highest levels of knowledge. However, gaps were identified in critical areas such as advanced airway management, cardiac arrest recognition, and understanding of aviation physiology, particularly cabin pressurization and decompression sickness. Additionally, 2125 (42.5%) of the participants expressed the need for further training in managing in-flight emergencies, citing concerns about unfamiliarity with specific emergencies and medicolegal implications.

Conclusion: While overall knowledge levels were relatively high, the study highlights significant gaps in critical areas of in-flight emergency management. These findings underscore the need for targeted educational interventions and training programs focused on the unique challenges of the aviation environment. Addressing these gaps will enhance the preparedness and confidence of healthcare professionals, ultimately improving patient outcomes during air travel.

## Introduction

The prevalence of air travel in recent years has inevitably led to an increase in in-flight medical emergencies (IFMEs), and it is important to assess the preparedness of healthcare providers to respond effectively to such situations [1]. In these services, emergency medicine, family medicine, and intensive care unit (ICU) physicians are frequently called upon to provide medical assistance during air travel [2]. However, their readiness, reliability, and behavior in managing IFMEs, especially in the Kingdom of Saudi Arabia (KSA), have not been thoroughly studied. 

In addition to the direct impact on passengers' health and safety, IFMEs also have implications for airlines, crew members, and the overall travel experience [3]. Prompt and proficient medical intervention can mitigate risks, alleviate passenger distress, and prevent disruptions to flight schedules. Conversely, inadequate management of IFMEs may lead to adverse outcomes, including fatalities, legal implications, and reputational damage to airlines [4]. 

IFMEs represent a unique challenge due to complex flight conditions and limited resources available for treatment [5]. These emergencies can range from minor issues such as seizures, convulsions, cardiac arrest, and stroke, to severe cases [3]. With millions of individuals flying each year, the potential for IFMEs is enormous, underscoring the importance of ensuring that healthcare providers are prepared to adequately deal with such situations [6]. The expertise required to handle an IFME goes beyond the training of the cabin crew, who are typically skilled in managing minor complaints like mild fever, giddiness, or minor trauma [7]. Many medical professionals do not possess the specific training required to diagnose or handle medical emergencies that occur during flights. However, they may find themselves morally obligated to intervene in such situations, even when they least anticipate it [8,9]. 

In addition to the direct impact on passenger health and safety, IFMEs also have implications for airlines, employees, and the overall travel experience [10]. Early and effective medical intervention can reduce risks, reduce passenger suffering, and prevent disruption of flight plans. The burden of IFME treatment extends beyond the immediate impact on individuals on board. It details challenges affecting the airline, passengers, and healthcare systems as a whole, with overlapping implications across the air transportation system. A comprehensive retrospective analysis conducted on a specific airline revealed that one in 11,000 passengers experienced an emergency situation during their flight [11]. 

Passengers with preexisting conditions, such as those with cardiopulmonary issues, may experience worsened symptoms. Factors like anxiety, fear of flying, claustrophobia, turbulence, and take-off or landing can further increase the chances of unexpected health deterioration. In addition to these factors, the general stressors of travel, such as lack of sleep, jet lag, digestive problems, missing medication doses, and increased risk of blood clots, all contribute to a higher likelihood of passengers experiencing a medical emergency during longer flights [8,12]. 

IFMEs occur in an unpredictable aviation environment, characterized by limited locations and limited infrastructure [13]. This unique situation poses significant challenges in dealing with mid-flight medical emergencies. From sudden cardiac arrest to acute respiratory distress and obstetric complications, IFMEs provide a variety of medical conditions that require a wide range of applicable medical responses to the aviation environment surroundings including limited medical equipment and space further enhancing the delivery of complex and effective care. Furthermore, the impact of IFME extends beyond the immediate health concerns of affected passengers. IFMEs can disrupt flight operations, causing flight delays, diversions, or even emergency landings [14]. Such problems not only cause passenger inconvenience but also cost airlines in terms of fuel, crew costs, and potential compensation for affected passengers. Furthermore, flight attendants have to deal with IFMEs to balance other responsibilities, increasing the strain on resources and personnel. 

Effective and timely medical services in IFME are essential not only to reduce health risks but also to preserve the overall travel experience. Prompt treatment can ease passenger distress, reassure fellow passengers, and prevent disruption to flight plans. Conversely, an inadequate or missing IFME program can have adverse consequences, including mortality, which can have a significant impact on affected individuals and their families. 

From a legal perspective, airlines may face potential liability issues if IFMEs are not properly handled. Failing to provide adequate medical assistance or being negligent in responding to an emergency can expose airlines to lawsuits and legal consequences. Furthermore, incidents involving poorly maintained IFMEs can damage the reputation of airlines, affecting customer trust and loyalty. 

The study aims to identify specific gaps in training, resources, and policies that may hinder the effective management of IFMEs from the perspective of healthcare providers. This could be a lack of access to knowledge, skills, or resources. By identifying these areas, targeted interventions can be designed to increase the preparedness of health professionals in the management of IFME.

## Materials and methods

The present study employed a web-based descriptive cross-sectional design to assess the knowledge, confidence, and attitudes of emergency medicine, family medicine, and ICU doctors in Saudi Arabia regarding IFMEs in the period between June and October 2024. 

The sample size for this study was calculated to ensure adequate statistical power to detect significant differences in knowledge levels among physicians in Saudi Arabia. Using Cochran's formula for sample size determination: n = Z2⋅p⋅(1−p)/e2 where Z is the value for the desired confidence level (1.96 for 95% confidence), P is the estimated proportion of the population with adequate knowledge (assumed to be 50% for maximum variability), and the margin of error (set at 2%) resulting in a sample of 2401; however, we were able to collect 5000 responses so we included them in the study. The inclusion criteria included practicing emergency medicine, family medicine, and ICU doctors in Saudi Arabia who held valid medical licenses and had experience managing IFMEs. Doctors who were not currently practicing or had retired from clinical practice were excluded from the study. 

Data collection was conducted via an online survey distributed through various channels, including email invitations and direct face-to-face engagement by data collectors. The survey, designed in English and hosted on Google Forms, included sections that obtained informed consent, collected sociodemographic data, and assessed the participants' knowledge and attitudes toward IFMEs (Appendix A). The survey explored the participants' familiarity with common in-flight medical conditions and their ability to make rapid diagnostic decisions, administer appropriate treatments, and effectively communicate with flight crews. It also gathered information on the challenges and limitations perceived by the participants in managing such emergencies, along with their experiences and decision-making strategies. Upon collection, the data were managed by a team of five individuals who transferred the responses into an MS Excel (Microsoft Corporation, Redmond, Washington, United States), ensuring accuracy and completeness. The data were then imported into IBM SPSS Statistics for Windows, Version 26 (Released 2019; IBM Corp., Armonk, New York, United States) for statistical analysis. Descriptive statistics were used to summarize the data, with qualitative variables expressed as numbers and percentages, and quantitative data presented as mean and standard deviation (mean ± SD). The association between variables was assessed using the Chi-squared test (χ2), and a p-value of <0.05 was considered statistically significant. Subgroup analyses were performed based on participants' specialties, years of experience, and prior exposure to IFMEs. The study was conducted in accordance with ethical standards, with the research protocol receiving approval from the Institutional Review Board (IRB) of KFU-REC-2024-MAY ETHICS2267 on 8/05/2024 (Appendix B). The survey data were collected anonymously, and no identifying information, such as emails, was associated with the responses. Access to the data was restricted to the study investigators to maintain confidentiality and ensure the ethical integrity of the research process.

## Results

The study included 5,000 participants, with a mean age of 45.23 years (SD = 12.12). The sample consisted of 3,259 males (65.2%) and 1,741 females (34.8%). Participants were distributed across various regions: North (754, 15.1%), South (982, 19.6%), East (1321, 26.4%), Middle (1459, 29.2%), and West (484, 9.7%). Regarding specialties, 1531 (30.6%) of the participants were in emergency medicine, 1978 (39.6%) in family medicine, and 1491 (29.8%) in the ICU. The majority practiced in a community setting (2855, 57.1%), while the rest were in an academic setting (2145, 42.9%). The average number of years of clinical experience was 15.12 (SD = 8.1). Nearly half of the participants (2421, 48.4%) had received training on in-flight emergencies (Table 1). 

**Table 1:** Demographic characteristics of participants (n = 5,000)

Demographic characteristics (n = 5,000)	
Variable	n (%)
Age (years)	
Mean (SD)	45.23 (± 12.12)
Gender	
Male	3,259 (65.2%)
Female	1,741 (34.8%)
City of practice	
North	754 (15.1%)
South	982 (19.6%)
East	1,321 (26.4%)
Middle	1,459 (29.2%)
West	484 (9.7%)
Specialties	
Emergency medicine	1,531 (30.6%)
Family medicine	1978 (39.6%)
ICU	1,491 (29.8%)
Practice setting	
Academic setting	2,145 (42.9%)
Community setting	2,855 (57.1%)
Years of clinical experience	
Mean (SD)	15.12 (± 8.1)
Level of practice	
Resident	531 (10.7%)
Board eligible	742 (14.8%)
Board certified	2,124 (42.5%)
Consultant	1,321 (26.4%)
Fellowship	282 (5.6%)
Received training on in-flight emergencies	
Yes	2,421 (48.4%)
No	2,579 (51.6%)

In the assessment of clinical knowledge, the responses varied across different scenarios. About 2432 (48.6%) correctly responded to the scenario involving chest pain with a normal ECG, while 3125 (62.5%) identified the correct response for chest pain with a normal ECG but positive troponin. A significant majority (3634, 72.7%) recognized the correct approach in a cardiac arrest scenario, and 3325 (66.5%) correctly responded to advanced airway management and resuscitation algorithms. Notably, 4154 (83.1%) of the participants correctly identified the short-term relief for asthma patients, and 3521 (70.4%) were aware of the Global Initiative for Asthma Strategy 2021 (GINA 2021) guidelines for asthma treatment. For acute asthma with anaphylaxis/angioedema, 3276 (65.5%) knew the correct use of intramuscular (IM) epinephrine. Other responses included 4187 (83.7%) for identifying inhaled corticosteroids, 3654 (73.1%) for initial steps in status epilepticus, and 4246 (84.9%) for recognizing regular, painful uterine contractions leading to cervical effacement. Awareness of specific obstetric scenarios like shoulder dystocia also showed high correct response rates, with 3743 (74.9%) knowing the appropriate application of suprapubic pressure (Table 2).

**Table 2:** Participants' responses to clinical knowledge statements (n = 5,000) ECG: electrocardiogram; GINA 2021: Global Initiative for Asthma Strategy 2021

Clinical scenario	Correct response	
	Count	Percent
Chest pain, occurs at rest or progresses rapidly over a short period, normal ECG	2,432	48.6%
Chest pain, normal ECG, positive troponin	3,125	62.5%
Cardiac arrest, unconscious, unresponsive, no pulse, and no breathing	3,634	72.7%
Advanced airway management, pharmacology, and complex resuscitation algorithms	3,325	66.5%
Short-term relief for patients with asthma	4,154	83.1%
GINA 2021 guidelines: asthma treatment starts with inhaled corticosteroids	3,521	70.4%
IM epinephrine for acute asthma with anaphylaxis/angioedema	3,276	65.5%
Inhaled corticosteroids include mometasone, beclomethasone, ciclesonide	4,187	83.7%
Initial step in status epilepticus	3,654	73.1%
Important vital sign in refractory status epilepticus	3,231	64.6%
Regular, painful uterine contractions resulting in cervical effacement	4,246	84.9%
Spontaneous onset, vertex presentation, term delivery	4,187	83.7%
Lack of a palpable presenting part, palpation of a lower extremity	4,198	83.9%
Warning signs of shoulder dystocia	3,543	70.9%
Part of the baby involved in shoulder dystocia	3,232	64.6%
Part of the baby given suprapubic pressure	3,743	74.9%
McRoberts maneuver effectiveness in shoulder dystocia	3,538	70.8%

In terms of aviation physiology knowledge, a large majority of participants correctly identified key concepts. For instance, 3759 (75.2%) acknowledged that cabin pressure leads to a decrease in systemic oxyhemoglobin saturation, and 4124 (82.5%) correctly noted that cabin air has lower humidity than at ground level. However, only 2315 (46.3%) correctly understood that commercial airplane cabins are typically pressurized to sea-level altitude. The knowledge about the expansion of gas in body cavities at low cabin pressure was correctly identified by 3532 (70.6%) of the participants. Awareness regarding altitude restrictions for patients with acute asthma exacerbation was noted in 3258 (65.2%) of responses. Additionally, 3175 (63.5%) of the participants recognized the risk of wound dehiscence or bowel perforation postabdominal surgery with air travel, and 4231 (84.6%) were aware of the increased venous thromboembolism risk associated with long-haul flights. Some misconceptions were evident, such as only 2512 (50.2%) correctly identifying confusion as the most common symptom of decompression sickness and 1578 (31.6%) incorrectly believing cardiac arrest is the most common IFME. Nevertheless, 4259 (85.2%) accurately noted that only a minority of in-flight emergencies result in plane diversion (Table 3).

**Table 3:** Participants' responses to aviation physiology and related knowledge statements (n = 5,000)

Statement	True (%)		False (%)	
	Count	Percent	Count	Percent
Cabin pressure leads to a decrease in systemic oxyhemoglobin saturation	3,759	75.2%	1,241	24.8%
Low humidity in cabin air on commercial flights compared to ground level	4,124	82.5%	876	17.5%
Commercial airplane cabins are typically pressurized to sea-level altitude	2,315	46.3%	2,685	53.7%
Gas in body cavities can expand by 30% at low cabin pressure	3,532	70.6%	1,468	29.4%
Patients with acute asthma exacerbation benefit from altitude restriction	3,258	65.2%	1,742	34.8%
Risk of wound dehiscence or bowel perforation post abdominal surgery with air travel	3,175	63.5%	1,825	36.5%
Long haul flights are associated with increased venous thromboembolism risk	4,231	84.6%	769	15.4%
Most common symptom of decompression sickness is confusion	2,512	50.2%	2,488	49.8%
Air travel increases risk of preterm labor	2,756	55.1%	2,244	44.9%
Cardiac arrest is the most common in-flight medical emergency	1,578	31.6%	3,422	68.4%
Only a minority of in-flight emergencies result in plane diversion	4,259	85.2%	741	14.8%

Participants' confidence and attitudes toward managing IFMEs showed a range of perspectives. Only 918 (18.4%) of the participants strongly agreed that they would identify themselves as a doctor during an in-flight emergency, with 1985 (39.7%) agreeing and 1245 (24.9%) remaining neutral. On the contrary, 1546 (30.9%) disagreed with the idea of staying out if someone else offered assistance, and 387 (7.7%) strongly agreed with that sentiment. Regarding familiarity with the emergency, 1423 (28.5%) were neutral, while 1235 (24.7%) agreed they would not offer assistance if they were unfamiliar with the situation. Concerns about medicolegal implications were also notable, with 1532 (30.6%) agreeing that it affected their willingness to assist. A significant number of participants (2123, 42.5%) agreed that more training in managing IFMEs was necessary, and 27.5% strongly agreed. When assessing their training, 1750 (35%) agreed that their medical training provided adequate knowledge for in-flight emergencies, but only 543 (10.9%) strongly agreed with this statement. Confidence levels varied, with 1768 (35.4%) agreeing that they felt confident in responding to in-flight emergencies, while 387 (7.7%) strongly agreed (Table 4).

**Table 4:** Participants' confidence and attitude toward in-flight medical emergencies (n = 5,000)

Statement	Strongly disagree (%)	Disagree (%)	Neutral (%)	Agree (%)	Strongly agree (%)
I would identify myself as a doctor in an in-flight medical emergency	287 (5.7%)	565 (11.3%)	1,245 (24.9%)	1,985 (39.7%)	918 (18.4%)
I would stay out if someone else is offering assistance	985 (19.7%)	1,546 (30.9%)	1,128 (22.6%)	954 (19.1%)	387 (7.7%)
I would not offer assistance if unfamiliar with the emergency	746 (14.9%)	1,078 (21.6%)	1,423 (28.5%)	1,235 (24.7%)	518 (10.3%)
I am afraid of medicolegal implications	546 (10.9%)	1,023 (20.5%)	1,452 (29.0%)	1,532 (30.6%)	447 (9.0%)
I need more training in managing in-flight medical emergencies	198 (4.0%)	546 (10.9%)	759 (15.2%)	2,123 (42.5%)	1,374 (27.5%)
My medical training has given me adequate knowledge for in-flight emergencies	538 (10.7%)	1,046 (20.9%)	1,123 (22.5%)	1,750 (35%)	543 (10.9%)
My medical training is adequate for on-ground emergencies	259 (5.2%)	632 (12.6%)	1,128 (22.6%)	2,223 (44.5%)	758 (15.1%)
I feel confident responding to in-flight medical emergencies	523 (10.5%)	1,062 (21.2%)	1,259 (25.2%)	1,768 (35.4%)	388 (7.7%)
I understand the medical supplies on commercial airplanes	498 (9.9%)	1,038 (20.8%)	1,623 (32.5%)	1,485 (29.7%)	356 (7.1%)
I understand the aircrew's level of training for managing emergencies	485 (9.7%)	1,569 (31.4%)	1,612 (32.2%)	1,028 (20.6%)	306 (6.1%)
I understand the collaboration between aircrew, ground control, and doctors	508 (10.2%)	1,064 (21.3%)	1,589 (31.8%)	1,123 (22.5%)	716 (14.2%)

**Figure 1:**
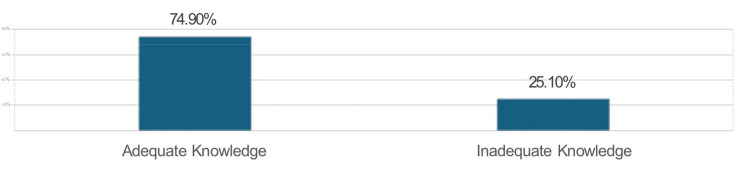
Assessment of knowledge level

Based on the participants' responses, 3745 (74.9%) demonstrated adequate knowledge of the topics assessed, while 1255 (25.1%) were categorized as having inadequate knowledge (Figure 1). 

The comparison between knowledge levels and demographic factors revealed significant differences. Males were more likely to have adequate knowledge (2504, 76.8%) compared to females (1242, 71.3%), with a significant p-value of 0.001. Participants practicing in the Middle region had the highest rate of adequate knowledge (1325, 90.8%), followed by those in the East (1111, 84.1%), South (752, 76.6%), North (498, 66.0%), and West (60, 71.4%) (p = 0.005). Regarding specialties, ICU specialists had the highest proportion of adequate knowledge (1200, 80.5%), followed by Family Medicine (1521, 76.9%) and Emergency Medicine (1025, 66.9%), with a highly significant p-value of 0.0001. These findings suggest that gender, region of practice, and specialty are associated with differences in knowledge levels among the participants (Table 5). 

**Table 5:** The association between the level of knowledge and demographic factors

Factors		Adequate knowledge (n = 3,746)		Inadequate knowledge (n = 1,254)		p-value
		Count	Percent	Count	Percent	
Gender	Male	2504	76.8%	755	23.2%	0.001
	Female	1242	71.3%	499	28.7%	
City of practice	North	498	66.0%	256	34.0%	0.005
	South	752	76.6%	230	23.4%	
	East	1111	84.1%	210	15.9%	
	Middle	1325	90.8%	134	9.2%	
	West	60	71.4%	24	28.6%	
Specialties	Emergency medicine	1025	66.9%	506	33.1%	0.0001*
	Family medicine	1521	76.9%	457	23.1%	
	ICU	1200	80.5%	291	19.5%	

## Discussion

This study aimed to evaluate the knowledge and practices of healthcare professionals regarding IFMEs and aviation physiology. The findings revealed significant insights into the demographic characteristics, clinical knowledge, aviation-related knowledge, and confidence levels of the participants, providing a comprehensive understanding of their preparedness for managing medical emergencies in a unique environment like an aircraft. The demographic data indicate a diverse sample with significant representation across different regions and specialties. Most participants were male, which is consistent with other studies that have observed a male predominance in certain medical fields, particularly in emergency medicine and ICU settings [15]. However, the variation in knowledge levels across different specialties and regions is noteworthy. Participants from the Middle region exhibited the highest level of adequate knowledge, followed by those from the East and South regions. This could be attributed to the availability of more comprehensive training programs or access to resources in these regions, which has been supported by previous research highlighting regional disparities in medical education. Additionally, ICU specialists demonstrated the highest knowledge levels, which aligns with their critical care training and frequent exposure to complex, high-stakes medical scenarios [16]. This is consistent with studies showing that critical care training significantly enhances clinicians' readiness to manage emergencies in various settings, including in-flight [17].

There were notable gaps in knowledge, particularly concerning the identification of cardiac arrest and the use of advanced airway management and pharmacology. Only 72.7% of the participants correctly identified the appropriate response to a cardiac arrest scenario, which is concerning given the critical importance of timely and accurate intervention in such cases. This finding aligns with research indicating that while clinicians are generally well-prepared for common medical emergencies, there are often deficiencies in their knowledge of advanced resuscitation techniques [18]. The moderate response rate for advanced airway management further highlights the need for more focused training in this area, particularly given its relevance to both in-flight and on-ground emergencies. 

Knowledge of aviation physiology and related issues varied significantly among participants. While most were aware of the effects of cabin pressure and low humidity on the human body, fewer participants correctly understood the typical cabin pressurization levels and the risks associated with gas expansion in body cavities. This suggests a gap in understanding the unique physiological challenges posed by the aviation environment, which could impact the effectiveness of medical interventions during flights. 

The misconception that commercial airplane cabins are pressurized to sea-level altitude, held by 53.7% of the participants, reflects a common misunderstanding that has been reported in other studies [19]. In reality, cabins are typically pressurized to an altitude equivalent to 6,000-8,000 feet above sea level, which can lead to significant physiological changes, particularly in patients with respiratory or cardiovascular conditions [20]. This gap in knowledge underscores the importance of incorporating aviation-specific training into medical education, particularly for clinicians who may be called upon to manage in-flight emergencies. 

Additionally, the findings related to decompression sickness and the risks associated with air travel following abdominal surgery highlight the need for greater awareness of these issues among healthcare professionals. The fact that only half of the participants correctly identified confusion as a common symptom of decompression sickness suggests that this area of knowledge is underemphasized in current training programs. Given the increasing frequency of air travel and the likelihood of encountering such scenarios, it is crucial to address these gaps to ensure that clinicians are fully prepared to manage all aspects of IFMEs. The participants' confidence in managing IFMEs varied, with a significant proportion expressing uncertainty about their ability to handle such situations effectively. 

Although a majority of participants indicated that they would identify themselves as a doctor in an in-flight emergency, a substantial minority were hesitant, citing concerns about medicolegal implications and unfamiliarity with the specific emergency. This finding is consistent with previous research indicating that concerns about legal liability are a significant barrier to clinicians' willingness to intervene in emergencies outside of traditional clinical settings [21,22]. The attitudes and practices of participants revealed a willingness to volunteer during IFMEs, albeit with notable apprehensions about their competence and potential legal issues. Most participants (76.7%) indicated they would probably or definitely volunteer to manage an IFME, demonstrating a strong sense of professional duty. Similar results are reported in the literature including the study of Chatfield et al. which reported that 42% mentioned that they had been asked to voluntarily assist to handle an IFME [23]. In addition, Alarifi et al. showed that 57.7 % of the participants would assist during an IFME [4]. However, only 16.8% felt fully confident in their ability to handle such situations, which is similar to the results of Ng et al. which showed that only 11.5% of the participating doctors felt confident to provide medical care during IFMEs [2]. These results highlight a critical gap in self-efficacy that could impact performance during actual emergencies [24,25]. 

The fact that 42.5% of participants agreed on the need for additional training in managing IFMEs further emphasizes the importance of addressing this issue. Training programs that specifically focus on the unique challenges of the aviation environment, including the limited availability of medical supplies and the need for collaboration with aircrew, could enhance clinicians' confidence and readiness to respond to in-flight emergencies [4,26]. Furthermore, improving understanding of the legal protections available to clinicians who assist in such situations could help alleviate concerns about medicolegal implications, thereby encouraging more active participation in managing IFMEs.

Implications for medical training and practice 

The findings of this study have important implications for medical training and practice, particularly in the context of preparing clinicians for the challenges of IFMEs. The significant knowledge gaps identified, particularly in the areas of advanced airway management, aviation physiology, and decompression sickness, suggest that current training programs may not be adequately addressing these critical areas. Incorporating more comprehensive training on these topics, including simulation-based exercises and case studies that reflect the realities of IFMEs, could help bridge these gaps and enhance clinicians' preparedness. 

Additionally, the study highlights the need for targeted interventions to improve knowledge levels among specific subgroups, such as clinicians practicing in regions with lower overall knowledge scores or those in specialties with less frequent exposure to emergency scenarios. Tailored educational programs that address the specific needs and challenges faced by these groups could help ensure that all clinicians, regardless of their practice setting or specialty, are adequately prepared to manage IFMEs. 

Limitations of the study

A few limitations must be acknowledged in this study. First, the data were collected through self-reported surveys, which may be subject to response bias, as participants might overestimate or underestimate their knowledge and confidence levels. Second, the cross-sectional design of the study captures a snapshot in time, limiting the ability to assess changes in knowledge or practices over time or in response to interventions. Additionally, the study was conducted among healthcare professionals from specific regions and specialties, which may not fully represent the broader population of medical practitioners. Lastly, the survey did not directly assess the participants' actual performance in IFMEs, which would provide a more objective measure of their competence. Despite these limitations, the findings provide valuable insights into the preparedness of healthcare professionals in managing IFMEs and highlight areas for further research and targeted training initiatives.

## Conclusions

In conclusion, while the overall knowledge levels among participants were relatively high, the identified gaps and variations in knowledge underscore the need for ongoing education and training to ensure that all healthcare professionals are equipped to respond effectively to medical emergencies in the unique environment of an aircraft. By addressing these gaps and enhancing the confidence and preparedness of clinicians, it is possible to improve patient outcomes and ensure the safety and well-being of passengers during air travel.

## Appendices

Appendix A

**Table 6:** The questionnaire of the study STEMI: ST-elevation myocardial infarction; NSTEMI: non-ST-elevation myocardial infarction; BLS: basic life support; ACLS: advanced cardiovascular life support; IV: intravenous; IM: intramuscular

Options/details	Question	Section
I agree to participate/I don’t agree to participate	Participation consent	Consent form
NA	Demographics	Section 1
Write text	Age	NA
Male/female	Gender	
North/South/East/Middle/West	City of practice	
Emergency medicine/family medicine/ICU	Specialties	
Academic/community	Practice setting	
Write text	Years of clinical experience	
Resident/board eligible/board certified/consultant/fellowship	Level of practice	
Yes/no	Training on in-flight medical emergencies	
Options	Statements	
Unstable angina/NSTEMI/STEMI	Chest pain, occurs at rest, or progresses rapidly over a short normal ECG	
Unstable angina/NSTEMI/STEMI	Chest pain, normal ECG, positive troponin	
Apply BLS protocol/apply ACLS protocol	Cardiac arrest, unresponsive, no pulse, no breathing or abnormal. CAP approach	
Apply BLS protocol/apply ACLS protocol	Advanced airway management, complex resuscitation algorithm ABCDE approach	
Proair/Ventolin/Xopenex/all of the above	Which of the below can provide short-term relief to patients with asthma	
True/false	Regarding GINA 2021 guidelines asthma treatment options start with inhaled corticosteroids	
True/false	IM epinephrine is indicated in addition to standard therapy for acute asthma associated with anaphylaxis and angioedema	
Mometasone/beclomethasone/ciclesonide/all of the above	Which of the following is an inhaled corticosteroid	
IV diazepam/IM lorazepam/airway + oxygen/phenytoin loading	The initial step to start with in the case of status epilepticus	
Temperature/oxygen saturation/pulse rate/gluco-check	Important vital sign to check in refractory status epilepticus	
Normal labor/abnormal labor/labor	Regular, painful uterine contractions, resulting in progressive cervical effacement and dilatation	
Normal labor/abnormal labor/labor	Criteria for normal labor (1) spontaneous at onset and at term (2) with vertex presentation (3) without undue prolongation	
Breech/cephalic	Lack of palpable presenting part, palpation of a lower extremity-usually the foot	
Turtle sign/slow progress/painful labor/all of the above	Warning signs of shoulder dystocia case	
Head/chest/anterior shoulder/posterior shoulder	Which part of the baby is involved in shoulder dystocia	
Ant. aspect of post. arm/post. aspect of ant. arm/ant. aspect of ant. arm	Where suprapubic pressure applies to	
90%/100%/70%	McRoberts maneuver resolves shoulder dystocia	
NA	Knowledge questions	Section 2
Aviation physiology		
True/false	Cabin pressure leads to a decrease in systemic oxyhemoglobin saturation	NA
True/false	The humidity in cabin air on a commercial airline flight is typically relatively low when compared to typical ground-level building interiors	
True/false	Commercial airplane cabins are typically pressurized to an altitude of sea level	
True/false	Gas in body cavities can expand by 30% at low cabin pressure associated with cruising attitudes. Medical conditions affected by air travel	
True/false	Patients with acute exacerbation of asthma benefit from altitude restriction	
True/false	Patients with recent abdominal surgery are at risk of wound dehiscence or bowel perforation with air travel	
True/false	Long-haul flights are associated with an increased risk of venous thromboembolism	
True/false	The most common symptom of decompression sickness, which can happen to scuba divers if they fly too soon after diving. There is confusion	
True/false	Air travel is associated with an increased risk of preterm labor	
Epidemiology		
True/false	Cardiac arrest is the most common in-flight medical emergency	NA
True/false	Only a minority of in-flight medical emergencies result in diversion of the plane	
Medicolegal issues		
True/false	Medical doctors, who are passengers on commercial airplanes, are obligated legally to respond to in-flight medical emergencies in Malaysia	NA
True/false	For flights in international airspace, the country where the aircraft is registered has legal jurisdiction on whether medical doctors are legally obligated to provide assistance in in‐flight medical emergencies	
Equipment contents of an aircraft medical kit would typically include		
True/false	Sphygmomanometer	NA
True/false	Intravenous catheters	
True/false	Urinary catheter	
True/false	Laryngoscope	
Drug contents of an aircraft medical kit would typically include		
True/false	Epinephrine/adrenaline injectable	NA
True/false	Dextrose 50% injectable	
True/false	Oral aspirin	
True/false	Anticonvulsant injectable	
Support system		
True/false	Cabin crews are trained in basic life support	NA
True/false	Most airlines provide in‐flight medical consultation service with ground‐based physicians in the event of in-flight medical emergencies	
True/false	The responding physician on board has the final say on whether the plane will be diverted because of an in‐flight medical emergency	
NA	Confidence and attitude questions	Section 3
Disagree/strongly disagree/neutral/agree/strongly agree	I would identify myself as a doctor and offer assistance in the event of an in‐flight medical emergency	NA
Disagree/strongly disagree/neutral/agree/strongly agree	I would stay out of an in‐flight medical emergency if someone else is already offering their assistance	
Disagree/strongly disagree/neutral/agree/strongly agree	I would not offer assistance if I am not familiar with the nature of the emergency, even though I am the only healthcare professional on board	
Disagree/strongly disagree/neutral/agree/strongly agree	I am afraid of the medicolegal implications which may arise from my assistance in an in‐flight medical emergency	
Disagree/strongly disagree/neutral/agree/strongly agree	I need more training in managing in‐flight medical emergencies	
Disagree/strongly disagree/neutral/agree/strongly agree	My medical training has given me adequate knowledge and skills to render assistance during an in‐flight medical emergency	
Disagree/strongly disagree/neutral/agree/strongly agree	My medical training has given me adequate knowledge and skills to render assistance during a medical emergency on the ground level	
Disagree/strongly disagree/neutral/agree/strongly agree	I feel confident responding to an in‐flight medical emergency and providing competent care	
Disagree/strongly disagree/neutral/agree/strongly agree	I have an adequate understanding of what medical supplies are available on commercial airplanes	
Disagree/strongly disagree/neutral/agree/strongly agree	I have an adequate understanding of the level of training of commercial aircrew in managing in‐flight medical emergencies	
Disagree/strongly disagree/neutral/agree/strongly agree	I have an adequate understanding of how the aircrew, ground-based medical control, and the onboard volunteer healthcare provider collaborate to manage an in‐flight medical emergency	

Appendix B

https://drive.google.com/file/d/1FoWnvaDRYlol18qWbzRcOeOLv2zr7Fcg/view?usp=drivesdk
